# Circulation of multiple serotypes of highly divergent enterovirus C in the Xinjiang Uighur Autonomous Region of China

**DOI:** 10.1038/srep33595

**Published:** 2016-09-19

**Authors:** Yong Zhang, Qiang Sun, Hui Cui, Dongmei Yan, Qin Fan, Yang Song, Shuangli Zhu, Xiaolei Li, Guohong Huang, Tianjiao Ji, Lan Hu, Dongyan Wang, Qian Yang, Wenbo Xu

**Affiliations:** 1 WHO WPRO Regional Polio Reference Laboratory and Key Laboratory of Medical Virology and Viral Diseases, National Health and Family Planning Commission of China, National Institute for Viral Disease Control and Prevention, Chinese Center for Disease Control and Prevention, Beijing, People’s Republic of China; 2 Xinjiang Uighur Autonomous Region Center for Disease Control and Prevention, Urumqi City, Xinjiang Uighur Autonomous Region, People’s Republic of China

## Abstract

Poliomyelitis associated with circulating vaccine-derived polioviruses (cVDPVs) is a serious public health issue in the post-eradication era, and the occurrence of recombinant cVDPVs emphasizes the need to elucidate enterovirus C (EV-C) epidemiology. Stool samples were collected from 826 healthy children in Southern Xinjiang in 2011 to investigate EV-C circulation and epidemiology. Thirty-six EV-Cs were isolated and assigned to eight EV-C serotypes by molecular serotyping, suggesting the circulation of diverse EV-Cs in Xinjiang. Phylogenetic analysis showed that the Xinjiang EV-C strains had larger variation compared to the prototype and other modern strains. Additionally, the results showed unique characteristics of Xinjiang EV-Cs, such as the cytopathicity of CV-A1 strains to RD cells; the high divergence in CV-A11, CV-A13, CV-A17, and CV-A20 strains; the divergence of Xinjiang CV-A24 from AHC-related CV-A24 variant stains distributed worldwide; and the circulation of two novel EV-C serotypes (EV-C96 and EV-C99). Evaluations of this dense and diverse EV-C ecosystem will help elucidate the processes shaping enteroviral biodiversity. This study will improve our understanding of the evolution of enteroviruses and the recombination potential between polioviruses and other EV-Cs.

Enterovirus C (EV-C) is the type species of the genus *Enterovirus* in the family *Picornaviridae* and order *Picornavirales*^1^. The species EV-C comprises 23 serotypes: poliovirus (PV: serotype 1, 2, and 3), coxsackievirus group A (CV-A: serotype 1, 11, 13, 17, 19–22, and 24), and several recently identified novel EV serotypes, designated EV-C95–C96^2^,^3^, EV-C99^4^,^5^, EV-C102^3^, EV-C104–C105^6^,^7^, EV-C109^8^, EV-C113^9^, and EV-C116–C118^7^,^10^,^11^. EV-C viruses are the causative agents of the common cold, acute flaccid paralysis (AFP), acute hemorrhagic conjunctivitis, herpangina, and other illnesses^12^,^13^.

Enteroviruses are small, non-enveloped viruses comprising 60 copies each of the capsid proteins VP4, VP2, VP3, and VP1, and a positive-sense, single-stranded RNA genome. The viral RNA (approximately 7450 nucleotides) contains a long open reading frame flanked by a 5′-untranslated region (UTR) and a 3′-UTR. The 5′-UTR, approximately 740 nucleotides in length, contains an internal ribosome entry site (IRES) that is involved in replication and internal initiation of translation of the genomic RNA^14^. A single polyprotein translated from the RNA strand is first cleaved into three polyprotein precursors: P1, P2, and P3. P1 is processed to yield 4 structural proteins, VP1–VP4; P2 and P3 are precursors of the nonstructural proteins 2A–2C and 3A–3D, respectively.

Poliomyelitis associated with mutated and pathogenic poliovirus strains derived from the oral polio vaccine has been a serious public health issue in the post-eradication era especially in polio free countries such as China^15^,^16^. Such pathogenic circulating vaccine-derived polioviruses (cVDPVs) have been reported in many poliomyelitis outbreaks in various countries, including China^17^,^18^,^19^,^20^. Most cVDPVs are recombinants containing unidentified EV-C donor sequences in their non-structural region^21^,^22^,^23^,^24^. The role of recombination in the generation and function of recombinant cVDPVs remains unclear, although some studies have shown that EV-C donor sequences affect specific characteristics of the cVDPVs; in particular, the EV-C-derived 3′ ends of cVDPV genomes have been associated with cVDPV pathogenicity^25^. In addition, recombination between polio vaccine strains and non-polio enteroviruses depends on the presence in the infected host of viruses likely to recombine with poliovirus, such as EV-Cs, so recombination between VDPVs and EV-Cs may indicate the presence and frequency of these enteroviruses in the population and prolonged VDPV circulation in the community^26^. The occurrence of recombinant cVDPVs emphasizes the need to improve our knowledge of EV-C epidemiology.

Between July 3 and October 9, 2011, an outbreak of infection with imported, wild poliovirus occurred in the Xinjiang Uygur Autonomous Region of China, with 21 laboratory confirmed cases and 23 clinically compatible cases. A public health emergency was declared in Xinjiang after the outbreak was confirmed; the surveillance for AFP cases was enhanced. Five rounds of vaccination with oral polio vaccine (1^st^: August-September, 2011; 2^nd^: October, 2011; 3^rd^: November, 2011; 4^th^: March, 2012; and 5^th^: April, 2012) were conducted, and the outbreak was finally stopped 1.5 months after laboratory confirmation of the index case (25 August, 2011)^27^. Genetic recombination can play a role in the evolution of enteroviruses and has been reported to occur between wild polioviruses or polio vaccine strains and non-poliovirus EV-Cs^28^,^29^. However, little is known about the circulation and epidemiology of EV-C in the region where the outbreak occurred.

Poliovirus is a member of species EV-C and outbreaks of VDPVs (most contain EV-C donor sequences in their non-structural region) are of public health significance. In the present study, as part of the surveillance for wild poliovirus infections, 826 stool samples were collected from healthy children in Southern Xinjiang from August to December, 2011, meanwhile, the stool samples were used to investigate the circulation and epidemiology of potential VDPVs and other EV-Cs in this region where imported wild-type polioviruses had been detected. Molecular serotyping method was used to characterize the enteroviruses isolated in Xinjiang^30^,^31^. Thirty-six non-polio EV-C strains that belonged to eight serotypes were identified, indicating the circulation of diverse EV-Cs in Xinjiang. In addition, the molecular characteristics of the circulating Xinjiang EV-C isolates were investigated in more detail.

## Results

### Isolation of vaccine-related polioviruses in the healthy children in Southern Xinjiang

In this study, 826 stool samples were collected from healthy children (6 months to 16 years old) in Southern Xinjiang from August to December, 2011. 35 type I vaccine-related polioviruses, 7 type II vaccine-related polioviruses, 18 type III vaccine-related polioviruses, 5 type I and type II vaccine-related polioviruses mixtures, one type I, type II and type III vaccine-related polioviruses mixture were isolated and identified. Compared with corresponding serotypes of Sabin strains, the numbers of nucleotide substitutions of type I, type II, and type III vaccine-related polioviruses in *VP1* region were 0–6, 0–3, 0–4, respectively. No VDPVs were found in this study.

### Molecular serotyping and geographic distribution of other EV-Cs in Southern Xinjiang

**Figure 1:**
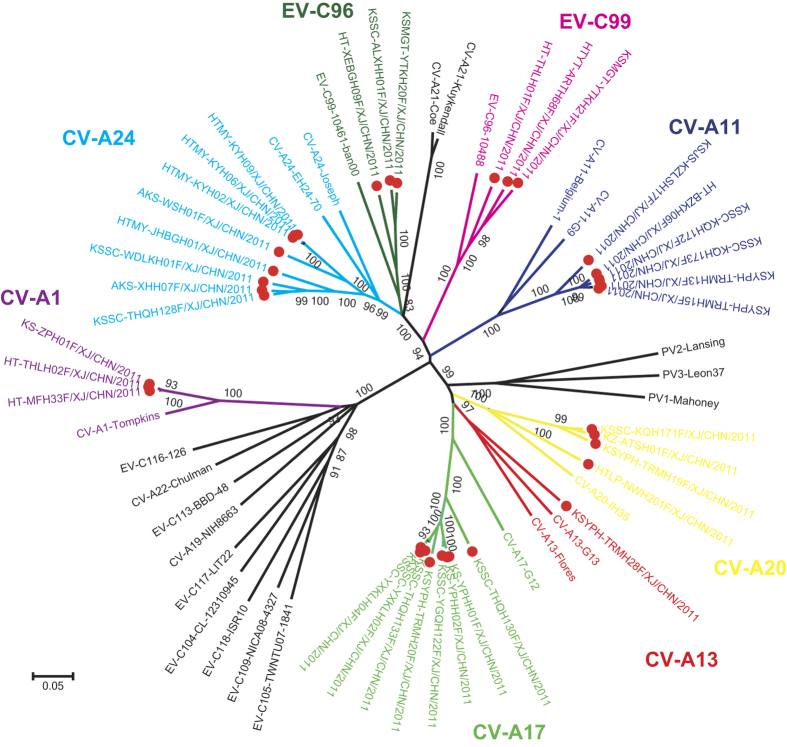
Phylogenetic tree of 36 EV-C strains collected in this study and all EV-C prototype strains based on complete *VP1* nucleotide sequences. The Xinjiang EV-C strains isolated in this study are indicated with red circles. The tree was constructed using the neighbor-joining method and Kimura 2-parameter model. The bootstrap support values were calculated from 1000 replicates, and values >80% are indicated at the main nodes.

In this study, 36 non-polio EV-C strains were isolated on either human rhabdomyosarcoma (RD) or human laryngeal epidermoid carcinoma (HEp-2) cell lines. Complete *VP1* sequences were acquired from all of the EV-C strains, and they showed 74.4–86.9% nucleotide identity and 87.5–96.1% amino acid identity (Table 1) with EV-C prototype sequences, which was in agreement with the molecular serotyping criteria previously defined^30^,^31^,^32^. A phylogenetic tree was constructed for all Xinjiang isolates and all EV-C prototype strains based on the complete *VP1* sequences (Fig. 1). The phylogeny indicated that all field isolates formed monophyletic groups with their respective homotypic prototype strains. This finding was supported by robust bootstrap values of 96–100%. These results allowed us to assign the 36 EV-C strains to eight serotypes (three CV-A1 strains, six CV-A11 strains, one CV-A13 strain, eight CV-A17 strains, four CV-A20 strains, eight CV-A24 strains, three EV-C96 strains, and three EV-C99 strains) (Fig. 1). CV-A1, CV-A11, CV-A13, and CV-A20 only grew in RD cells, EV-C99 only grew in HEp-2 cells, and CV-A17, CV-A24, and EV-C96 were able to grow in either RD or HEp-2 cell line (Table 1).

**Figure 2:**
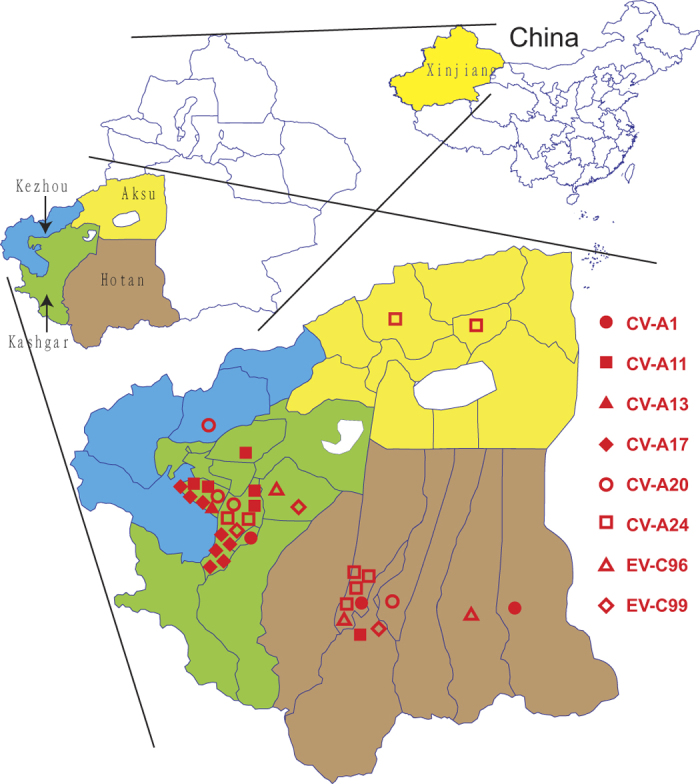
Geographic distribution of EV-C identified in southern Xinjiang. Thirty-six non-polio EV-C strains were isolated and identified. Eleven (two CV-A1, one CV-A11, one CV-A20, four CV-A24, two EV-C96, and one EV-C99) were isolated from Hotan prefecture, 22 (one CV-A1, five CV-A11, one CV-A13, eight CV-A17, two CV-A20, two CV-A24, one EV-C96, and two EV-C99) were isolated from Kashgar prefecture (255 samples), two CV-A24 were isolated from Aksu prefecture (31 samples), and one CV-A20 was isolated from Kezhou (65 samples) prefecture. The map was generated with MapInfo Pro software (version 11.0, http://www.pitneybowes.com/us/location-intelligence/geographic-information-systems/mapinfo-pro.html).

Among the strains, 11 EV-Cs (two CV-A1, one CV-A11, one CV-A20, four CV-A24, two EV-C96, and one EV-C99) were isolated from Hotan prefecture (from 475 samples total); 22 EV-Cs (one CV-A1, five CV-A11, one CV-A13, eight CV-A17, two CV-A20, two CV-A24, one EV-C96, and two EV-C99) were isolated from Kashgar prefecture (from 255 samples total); two CV-A24 were isolated from Aksu prefecture (from 31 samples total), and one CV-A20 was isolated from Kezhou prefecture (from 65 samples total) (Fig. 2).

### Sequence diversity in the *VP1* region of Xinjiang EV-C isolates

**Figure 3:**
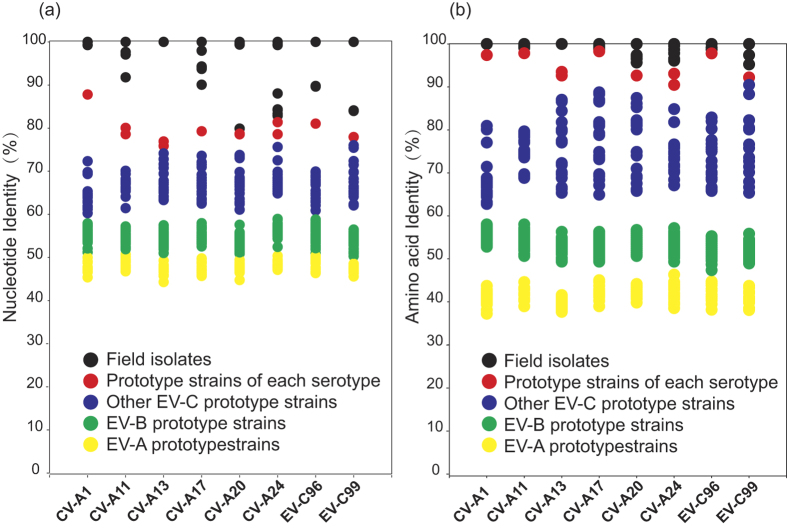
VP1 nucleotide and amino acid identity distribution plots. Nucleotide and amino acid identities were calculated for *VP1* sequence comparisons between 36 isolates and the corresponding prototype strain sequences using SigmaPlot 12.0 (Systat Software, Inc.). Similarity values were plotted separately for each of the 36 isolates. One field isolate of each EV-C serotype was selected for pairwise comparison in nucleotide identity distribution plots (**a**) and amino acid identity distribution plots (**b**), represented as 100%. The following EV-C field isolates were chosen for reference: CV-A1, strain HT-THLH02F; CV-A11, strain KSJS-KZLSH17F; CV-A13, strain KSYPH-TRMH28F; CV-A17, strain KSYPH-TRMH20F; CV-A20, strain KZ-ATSH01F; CV-A24, strain AKS-XHH07F; EV-C96, strain HTYT-ARTH68F; and EV-C99, strain KSMGT-YTKH20F. For CV-A11, CV-A13, and CV-A24, each virus has two prototype strains, so two plots were showed when filed isolates was compared with the prototype strains of these serotypes.

To describe the similarities between Xinjiang EV-C isolates and their corresponding prototype strains, nucleotide identity distribution plot and amino acid identity distribution plot were created, respectively. One field isolate of each EV-C serotype was selected for pairwise comparison in nucleotide identity distribution plots (Fig. 3a) and amino acid identity distribution plots (Fig. 3b), represented as 100%. Similar identity patterns were observed at both the nucleotide and amino acid levels; there was a clear genetic distinction between the Xinjiang EV-C isolates and the prototypes of the other two EV species, EV-A and EV-B (because EV-D has only five serotypes so far, it was not included in this analysis), which had sequence identities with those of the prototypes of EV-A and EV-B that ranged from 36.3–48.5% and from 30.3–45.8%, respectively (data not shown). In addition, the Xinjiang EV-C clinical isolates were most closely related to the corresponding prototypes, which had similarity identities ranging from 74.4% to 86.9% at the nucleotide level and from 87.5% to 96.1% at the amino acid level (Table 1).

**Figure 4:**
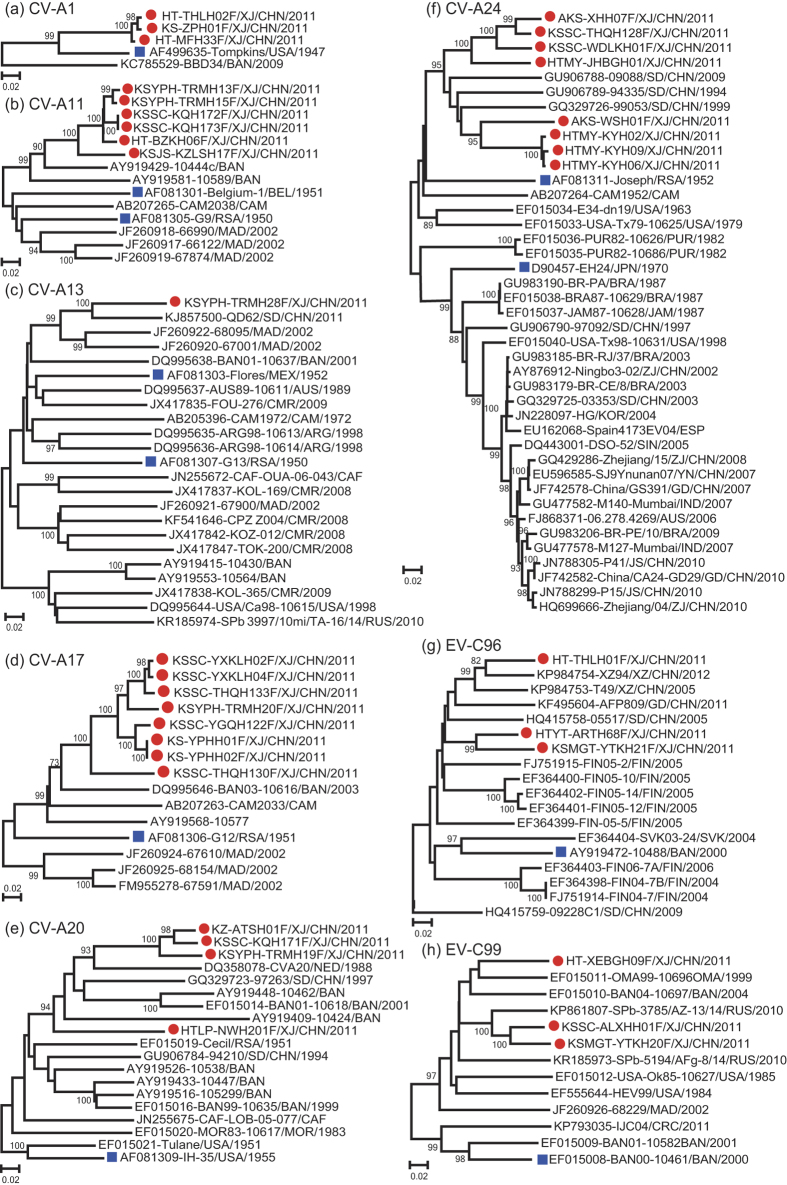
Phylogenetic relationships of Xinjiang EV-C isolates. (**a**) CV-A1, (**b**) CV-A11, (**c**) CV-A13, (**d**) CV-A17, (**e**) CV-A20, (**f**) CV-A24, (**g**) EV-C96, and (**h**) EV-C99. Phylogenetic trees were constructed for each serotype by the neighbor-joining method implemented in MEGA 5.0. Filled red circles label Xinjiang EV-C isolates, while filled blue squares label the corresponding prototype strains.

In addition, we evaluated the relationships between the EV-C strains circulating in Xinjiang in 2011 and those isolated in other locations by aligning the complete *VP1* region of the 36 Xinjiang EV-C isolates with those of clinical isolates and prototype strains available in the GenBank database using phylogenetic software. Figure 4 displays the dendrogram obtained from this nucleotide alignment. The results showed that the Xinjiang EV-C isolates were genetically distinct from strains isolated elsewhere.

### Xinjiang CV-A1 strains were cytopathic to human RD cells

Previous studies have shown that CV-A1 can only be isolated from suckling mice and cannot be isolated from cell lines^33^. However, three CV-A1 strains (HT-THLH02F, HT-MFH33F, and KS-ZPH01F) isolated in this study were found to be cytopathic to RD cells and to produce typical cytopathic effects (CPE) 4 to 5 days post-inoculation. Molecular serotyping revealed that these strains belonged to the CV-A1 serotype; they clustered with the prototype CV-A1 strain in the phylogeny of *VP1* coding region sequences (Fig. 4a). These three CV-A1 strains had high nucleotide and amino acid similarities (99.4% and 98.9%) to one another, while their average nucleotide and amino acid similarities compared to the prototype strain were 86.75% and 95.25%, respectively (Table 1).

### High sequence divergence of Xinjiang CV-A11, CV-A13, CV-A17 and CV-A20 strains

Xinjiang CV-A11, CV-A13, CV-A17, and CV-A20 strains were identified as highly divergent viruses because the intratypic differences in their *VP1* sequences compared to the corresponding prototypes strains and other clinical isolates (similarities ranged from 71.0% to 87.5% on the nucleotide level). These sequences approached the 25% nucleotide divergence limit for grouping within a serotype (Table 1). All of the prototype strains of the four EV-C serotypes were discovered in the 1950s, and, even though the prototype strains were isolated many years ago, there are still not many complete *VP1* sequences available in GenBank.

Unlike CV-A11 (six strains), CV-A17 (eight strains), and CV-A13 (one strain), which had high similarities within serotypes, the nucleotide sequence of CV-A20 (four strains) was more divergent. One of the strains, strain HTLP-NWH201F, showed higher divergence compared to the other three CV-A20 strains, indicating there were at least two independent lineages of CV-A20 circulating in Xinjiang (Fig. 4b–e). From the point of view of complete *VP1* nucleotide sequence alignment of CV-A20 with prototype strain sequences, all four Xinjiang CV-A20 strains had a three-nucleotide insertion at site 69–71 (*VP1* region) compared with the prototype strain (strain IH35, GenBank number, AF499642), but the inserted bases are different. Strain HTLP-NWH201F has an AAC insertion that leads to an asparagine insertion at site 24 (VP1 protein), while the other three Xinjiang CV-A20 strains have a UCU insertion that leads to a serine insertion. Strain HTLP-NWH201F has another AGU insertion in site 301–303 (*VP1* region) that leads to an additional serine insertion in site 101 (VP1 protein). Regardless of these differences, it was difficult to determine whether these viruses were continuously circulating indigenous strains or whether they represented periodic introductions of EV-Cs from outside the country.

### Xinjiang CV-A24 strains are highly divergent from other circulating strains that have caused acute hemorrhagic conjunctivitis (AHC) outbreaks

Acute hemorrhagic conjunctivitis (AHC) is a highly contagious, epidemic, eye disease that has been most commonly caused by an antigenic variant of CV-A24 (CV-A24v) over the past twenty years^13^,^34^,^35^. The *VP1* sequences of Xinjiang CV-A24 strains were compared with those of other CV-A24 strains obtained from other provinces in China or from other counties. The sequences of the eight Xinjiang CV-A24 strains clustered with CV-A24 strains isolated from AFP patients in Shandong province (GenBank nos GU906788, GU906789, and GQ329729), and were distinct from AHC-related strains of this serotype isolated elsewhere (Fig. 4f). The nucleotide and amino acid similarities of the *VP1* coding region sequences of the eight Xinjiang CV-A24 strains compared to the CV-A24 prototype strain were 77.3–79.2% and 91.1–93.4%, respectively. These strains showed 74.6–75.5% nucleotide and 91.1–93.1% amino acid identity to the previously described CV-A24v strains that caused AHC.

### Two novel EV-C serotypes (EV-C96 and EV-C99) were identified in Xinjiang

EV-C96 and EV-C99 are recently identified EV serotypes within the species EV-C. The prototype strains of EV-C96 (BAN00-10488) and EV-C99 (BAN00-10461) were isolated in Bangladesh in 2003 and in 2000, respectively. Three EV-C96 strains and three EV-C99 strains were identified in this study, and Xinjiang EV-C96 and EV-C99 strains showed relatively high nucleotide divergences from other EV-C96 and EV-C99 isolates available in GenBank (Fig. 4g,h). The *VP1* coding region of the three Xinjiang EV-C96 strains displayed 77.5–90.3% nucleotide and 90.0–96.4% amino acid identities to the previously described EV-C96 strains; and the *VP1* coding region sequence of the three Chinese EV-C99 strains displayed 73.2–88.9% nucleotide and 87.1–96.4% amino acid identity to the EV-C99 strains.

## Discussion

Recent research on cVDPVs bearing non-polio enterovirus sequences has shown that recombination of EV-C and vaccine-related strains or VDPV strains results in unexpected genetic diversity. There are many studies describing the epidemiology and evolution of EV-C strains worldwide^36^,^37^,^38^,^39^. Our results provide further evidence that EV-C is widely distributed, although EV-C isolates have been poorly studied due to the inability of certain serotypes to grow in common cell lines and the lack of specific antisera.

A lot of vaccine-related polioviruses were isolated among the 826 stool specimens screened in this study. This did not surprised us because many stool specimens were collected after the 1^st^ (August-September, 2011), 2^nd^ (October, 2011), and 3^rd^ rounds (November, 2011) supplementary OPV campaigns, especially in the third round OPV campaign were type I monovalent OPV, instead of the trivalent OPV that was used in the first two rounds OPV campaigns, this may be the cause of the higher number of type I vaccine-related poliovirus than that of type II and type III. And in addition to the vaccine-related polioviruses, another eight serotypes within the EV-C species have been discovered, indicating that this species has been continuously and widely circulating in Southern Xinjiang. Southern Xinjiang is in a warm temperate zone, which is suitable for enterovirus growth and survival.

The circulation of EV-C strains should be carefully monitored, because several outbreaks of poliomyelitis caused by cVDPVs containing sequences in the non-structural protein coding region from unidentified sources that are considered to be a result of recombination with EV-C have been documented^21^,^22^,^24^. Moreover, many recent studies have shown that such enterovirus species exist as a worldwide reservoir of genetic material, comprising a limited quantity of coding region sets defining a finite number of serotypes and a range of non-structural genes that recombine frequently to produce new virus variants^28^,^36^,^40^,^41^. Recombination between a slightly pathogenic vaccine poliovirus mutant and an EV-C strain may result in a recombinant that is more pathogenic, replicates to higher titers, and is maintained in circulation^23^,^36^,^42^.

Some EV-C (especially CV-A13, CV-A17, and CV-A20), which are similar to polioviruses at the nucleotide level and are known to recombine with vaccine polioviruses, are frequently found in countries such as the Philippines^22^, Madagascar^39^,^43^, Cambodia^26^, and Nigeria^36^, where cVDPVs are known to have resulted from recombination with EV-Cs. Recombination between two viruses depends on the co-infection of a single cell with both the viruses. Therefore, recombination between different types of vaccine polioviruses is possible in individuals receiving a multivalent polio vaccine^12^; however, recombination between polio vaccine strains and non-polio enteroviruses (NPEVs) depends on the presence of viruses likely to recombine with poliovirus, such as wild polioviruses or certain EV-Cs, in the infected host. The frequency of recombinant cVDPV emergence in vaccinated individuals and its subsequent circulation depends on the frequency of the recombination partners in the human population. In this sense, recombination between polio vaccine strains and non-vaccine enteroviruses is an indicator of the presence and frequency of these enteroviruses in the population, in addition to an indicator of the duration of viral circulation in the community^20^.

From the point of view of complete *VP1* nucleotide sequence alignment of CV-A20 with prototype strain sequences, all four Xinjiang CV-A20 strains have three-nucleotide insertions. Currently, there is an increasing focus on polymorphisms of the type short insertions and deletions (indels) in genomic research, indels will come to form an important source of genetic markers, easy and cheap to genotype, for studies of natural populations^44^; such indels serve as fingerprints for evolutionary pathways and indicate distance as well. Take one three-nucleotide insertion (site 69–71 in *VP1* region) as an example, the insertions in strain HTLP-NWH201F and the insertions in other three Xinjiang CV-A20 strains are very different and more than one single nucleotide substitution with more than one amino acid change would be required if there is a direct evolutionary relationship, this also supports that the nucleotide sequence of CV-A20 (four strains) was more divergent.

The number of the EV-C positive samples may have been underestimated by the cell lines used for viral isolation. Some enteroviruses that cannot grow in RD and HEp-2 cells may have been missed in this study. For instance, most of the newly identified EV-C such as EV-C104, -C105, -C109, -C113, -C116, C117, and -C118 were not reported by viral isolation in cell culture^7^,^8^,^9^,^10^,^45^,^46^,^47^,^48^. However, our research team tried to extract RNA directly from part of the stool suspensions, and screened such RNA with published pan EV-C primers (primer pairs 494 and 496; 495 and 497)^5^, and did not find above mentioned novel EV-Cs. Another pan EV-C primers used in this study (primer pairs EV-CY and EV-CZ) can amplify an 1.2 kb amplicons that cover the entire *VP1* region by a long distance PCR method, but it is not suitable for direct amplification of viral RNA from clinical samples because it is hard to amplify a large fragment when the copies of viral RNA in stool suspension is too low. It is possible to find more EV-Cs after the invention of the new sensitive pan EV-C primers that suitable for the direct detection of viral RNA from stool samples. In addition, next-generation sequencing technologies are revolutionising genomics and their effects are becoming increasingly widespread, commoditized and routine in recent years^49^, so next generation sequence analysis is very promising and provides a new method for identifying novel EV-C that cannot grow in the cell culture. Moreover, next generation sequence analysis of RNA from stool suspensions (and from viruses isolated in tissue culture) can yield full length genomic sequence data from culturable and uncuturable enteroviruses that can be used to reveal detailed information on enterovirus recombinations throughout the genome.

Nevertheless, strains of the eight EV-C serotypes analyzed in this study grew in at least one of these two cell lines. Interestingly, three CV-A1 strains that were cytopathic to RD cells were isolated in this study. Previous studies have shown that CA-A1, together with CV-A19 and CV-A22, are the enterovirus serotypes that have never successfully adapted to growth in cell culture^33^, instead requiring passage by intracranial inoculation of suckling mice. Our research team is currently using reverse genetic methods to elucidate the inherent mechanism of this phenomenon (the CPE seen in RD cells infected).

A distinct lineage of CV-A24, the so-called variant strains (CV-A24v), induced AHC^13^,^50^. In this study, Xinjiang CV-A24 isolates exhibited significant nucleotide divergence (20.8–28.2%) from the globally distributed CV-A24v strain, which is one of the major pathogens that causes AHC (other pathogens include enterovirus D70, and some serotypes of human adenoviruses). Based on the phylogenetic analysis, Xinjiang CV-A24 isolates clustered with the CV-A24 strains isolated from AFP patients in Shandong province of China, indicating that the Xinjiang strains and Shandong strains had a common origin. Although Xinjiang CV-A24 strains were isolated from a healthy population, their pathogenic potential cannot be excluded. Further surveillance is required to ascertain if the Xinjiang CV-A24 isolates can cause AHC.

In summary, an investigation of healthy children to identify enteroviruses circulating in Southern Xinjiang where poliomyelitis cases occurred revealed the presence of highly diverse EV-C strains of different serotypes in this region. Thirty-six EV-C strains were isolated from 826 healthy children in southern Xinjiang in the first large-scale survey to determine the EV prevalence among young children in Xinjiang. The results showed a complex and special EV-Cs ecosystem, with Xinjiang CV-A1 strains that were cytopathic to RD cells, highly divergent Xinjiang CV-A11, CV-A13, CV-A17, and CV-A20 strains, the divergence of Xinjiang CV-A24 from the widely distributed AHC-related CV-A24v stain, and the circulation of two novel EV-C serotypes (EV-C96 and EV-C99). We provide evidence for the circulation of EV-C in Southern Xinjiang where an outbreak of imported wild poliovirus had occurred. Circulating EV-Cs can help to elucidate the processes shaping enteroviral biodiversity. Data from this study provide valuable information about putative donor sequences to cVDPVs. These results will also contribute to an understanding of the evolution of enteroviruses and recombination between polioviruses and other EV-Cs.

## Materials and Methods

### Ethics Statement

This study did not involve human participants or human experimentation. The only human materials used were stool samples collected from healthy children at the instigation of the Ministry of Health, P. R. of China, for public health purposes, and written informed consent for the use of their clinical samples was obtained from the parents of the children whose samples were analyzed. This study was approved by the second session of the Ethics Review Committee of the National Institute for Viral Disease Control and Prevention, Chinese Center for Disease Control and Prevention, and the methods were carried out in accordance with the approved guidelines.

### Viral isolation

The EV-C strains used in this study are listed in Table 1. The virus strains were isolated from 826 stool specimens collected from 826 health children in the Hotan (475 samples), Kashgar (255 samples), Aksu (31 samples), and Kezhou (65 samples) prefectures in Xinjiang, China, in 2011. The stool samples were processed according to standard protocols recommended by WHO^51^. RD and HEp-2 cell lines were used for virus isolation. All viruses were isolated from original specimens after two passages in RD and HEp-2 cells. These two cell lines were obtained from the WHO Global Poliovirus Specialized Laboratory USA and were originally purchased from the American Type Culture Collection.

### Molecular typing and determination of the complete VP1 nucleotide sequence

Viral RNA was extracted from infected cell culture supernatants that produced typical CPE in RD or HEp-2 cells using a QIAamp Viral RNA Mini Kit (Qiagen, Valencia, CA, USA). The primer pair EVP4/OL68-1 was used to amply the *VP4* region of the viruses, and RT-PCR was performed with an Access RT-PCR Kit (Promega, Madison, Wisconsin, USA) according to the manufacturer’s instructions. The PCR products obtained were purified using a QIAquick Gel Extraction Kit (Qiagen), and the amplicons were bi-directionally sequenced using an ABI PRISM 3130 Genetic Analyzer (Applied Biosystems, Hitachi, Japan). EV-C species was determined using the online Enterovirus Genotyping Tool (http://www.rivm.nl/mpf/enterovirus/typingtool)^52^. Then, the complete *VP1* region of each EV-C strain was amplified using the universal primer set EV-CY/EV-CZ or serotype specific primers (Table 2). EV-C serotypes were also determined using the online Enterovirus Genotyping Tool^52^.

### Phylogenetic and bioinformatics analyses

The nucleotide and deduced amino acid sequences of the 36 Xinjiang EV-C isolates were compared to one another and to those of other EV-A, EV-B, and EV-C prototype strains by pairwise alignment using the MEGA program (version 5.05; Sudhir Kumar, Arizona State University, Tempe, Arizona, USA)^53^, and identity matrices were analyzed by plotting VP1 amino acid identities versus *VP1* nucleotide identities for each virus pair using the SigmaPlot program (version 10.0; Systat Software Inc., San Jose, CA, USA). The phylogenetic trees of Xinjiang EV-C sequences obtained in this study and EV-C sequences downloaded from the GenBank database was constructed by the neighbor-joining method based on the Kimura 2-parameter model with majority-rule consensus with 1000 bootstrap replicates shown as percentages. Bootstrap values greater than 80% were considered statistically significant for grouping. The map in Fig. 2 was generated with MapInfo Pro software (version 11.0, http://www.pitneybowes.com/us/location-intelligence/geographic-information-systems/mapinfo-pro.html).

### Nucleotide sequence accession numbers

The 36 complete *VP1* nucleotide sequences of eight serotypes of EV-C strains that were determined in this study were deposited in the GenBank database under the accession numbers JX174176–JX174177, KF129411–KF129412, and KU531400–KU531431.

## Additional Information

**How to cite this article**: Zhang, Y. *et al*. Circulation of multiple serotypes of highly divergent enterovirus C in the Xinjiang Uighur Autonomous Region of China. *Sci. Rep.* **6**, 33595; doi: 10.1038/srep33595 (2016).

## Figures and Tables

**Table 1:** Molecular typing based on the complete *VP*1 sequences of Xinjiang isolates.

Isolates			Highest identity with the prototype (%)			Accession no.
	Location^1^	Cell line	Serotype	nucleotide	Amino acid	
HT-THLH02F/XJ/CHN/2011	HT	RD	CV-A1	86.7	95.6	JX174176
KS-ZPH01F/XJ/CHN/2011	KS	RD	CV-A1	86.8	94.9	JX174177
HT-MFH33F/XJ/CHN/2011	HT	RD	CV-A1	86.9	95.6	KU531400
KSJS-KZLSH17F/XJ/CHN/2011	KS	RD	CV-A11	77.2	95.4	KU531401
KSYPH-TRMH13F/XJ/CHN/2011	KS	RD	CV-A11	77.5	95.7	KU531402
KSYPH-TRMH15F/XJ/CHN/2011	KS	RD	CV-A11	77.6	96.1	KU531403
KSSC-KQH172F/XJ/CHN/2011	KS	RD	CV-A11	77.6	95.4	KU531404
KSSC-KQH173F/XJ/CHN/2011	KS	RD	CV-A11	77.6	95.4	KU531405
HT-BZKH06F/XJ/CHN/2011	HT	RD	CV-A11	78.1	95.7	KU531406
KSYPH-TRMH28F/XJ/CHN/2011	KS	RD	CV-A13	74.4	90.3	KU531407
KSYPH-TRMH20F/XJ/CHN/2011	KS	RD	CV-A17	77.7	94.8	KU531408
KSSC-THQH133F/XJ/CHN/2011	KS	RD	CV-A17	77.6	95.4	KU531409
KSSC-YGQH122F/XJ/CHN/2011	KS	RD	CV-A17	78.5	95.8	KU531410
KSSC-THQH130F/XJ/CHN/2011	KS	RD	CV-A17	78.6	95.1	KU531411
KSSC-YXKLH02F/XJ/CHN/2011	KS	RD	CV-A17	77.2	94.4	KU531412
KSSC-YXKLH04F/XJ/CHN/2011	KS	RD	CV-A17	77.1	95.1	KU531413
KS-YPHH01F/XJ/CHN/2011	KS	HEp-2	CV-A17	78.1	95.4	KU531414
KS-YPHH02F/XJ/CHN/2011	KS	HEp-2	CV-A17	78.1	95.4	KU531415
KZ-ATSH01F/XJ/CHN/2011	KZ	RD	CV-A20	78.3	92.8	KU531416
KSYPH-TRMH19F/XJ/CHN/2011	KS	RD	CV-A20	77.2	92.1	KU531417
KSSC-KQH171F/XJ/CHN/2011	KS	RD	CV-A20	77.7	93.1	KU531418
HTLP-NWH201F/XJ/CHN/2011	HT	RD	CV-A20	77.4	91.5	KU531419
AKS-XHH07F/XJ/CHN/2011	AKS	RD	CV-A24	77.9	91.8	KU531420
KSSC-THQH128F/XJ/CHN/2011	KS	RD	CV-A24	79.2	93.4	KU531421
HTMY-JHBGH01/XJ/CHN/2011	HT	RD	CV-A24	78.9	93.1	KU531422
AKS-WSH01F/XJ/CHN/2011	AKS	HEp-2	CV-A24	75.7	92.1	KU531423
HTMY-KYH06/XJ/CHN/2011	HT	HEp-2	CV-A24	77.6	91.5	KU531424
HTMY-KYH02/XJ/CHN/2011	HT	HEp-2	CV-A24	77.5	91.5	KU531425
HTMY-KYH09/XJ/CHN/2011	HT	HEp-2	CV-A24	77.3	91.1	KU531426
KSSC-WDLKH01F/XJ/CHN/2011	KS	RD	CV-A24	78.0	92.5	KU531427
HTYT-ARTH68F/XJ/CHN/2011	HT	HEp-2	EV-C96	79.6	93.2	KU531428
HT-THLH01F/XJ/CHN/2011	HT	RD	EV-C96	79.5	93.2	KU531429
KSMGT-YTKH21F/XJ/CHN/2011	KS	RD	EV-C96	79.4	92.2	KU531430
KSMGT-YTKH20F/XJ/CHN/2011	KS	HEp-2	EV-C99	76.1	90.8	KU531431
HT-XEBGH09F/XJ/CHN/2011	HT	HEp-2	EV-C99	76.4	87.5	KF129411
KSSC-ALXHH01F/XJ/CHN/2011	KS	HEp-2	EV-C99	75.8	88.8	KF129412

^1^HT: Hotan prefecture; KS: Kashgar prefecture; KZ: Kezhou prefecture; AKS: Akesu prefecture.

**Table 2:** Primers used in this study.

Primer	Nucleotide position (nt)	Primer sequence (5′-3′)	Orientation	Reference
EV-P4	541–560	CTACTTTGGGTGTCCGTGTT	Forward	54
OL68-1	1178–1197	GGTAAYTTCCACCACCANCC	Reverse	54
EV-CY	2296–2315	ATGYTIGGIACICAYITIATHTGGGA	Forward	This study
EV-CZ	3472–3491	ATRTTRCADATYTTGTAICCIGC	Reverse	This study
CV-A1-VP1-S	2345–2364	CCAGTGGGACTCCAGTAACC	Forward	This study
CV-A1-VP1-A	3472–3491	CCTTGAGCACCACTCTCACA	Reverse	This study
CV-A11-VP1-S	2420–2439	CCACCACAATGACAATGCTC	Forward	This study
CV-A11-VP1-A	3458–3477	CGCTGTGTAAACTGCCTTGT	Reverse	This study
CV-A13-VP1-S	2293–2312	TTACCGTCGCACTGTGAAAG	Forward	This study
CV-A13-VP1-A	3469–3488	AGTCTTCTGGAGTGGCCAAA	Reverse	This study
CV-A17-VP1-S	2300–2319	ATCAGCAACACAGCATACCG	Forward	This study
CV-A17-VP1-A	3553–3572	AGTCTGTGCCCTGTGCTTTG	Reverse	This study
CV-A20-VP1-S	2255–2277	TGATTTTACAGAAGGGGGTTACA	Forward	This study
CV-A20-VP1-A	3441–3460	CAACAGGTCTCTGTTCCACA	Reverse	This study
CV-A24-VP1-S	2402–2421	CCAACATCAATGTTCATGCT	Forward	This study
CV-A24-VP1-A	3539–3558	CACCATCAGGTCTCTGTTCC	Reverse	This study
EV-C96-VP1-S	2416–2435	TGTCCTGATTTCTCAGTGCG	Forward	This study
EV-C96-VP1-A	3514–3533	AGGAAATCTCTGTTCCACAT	Reverse	This study
EV-C99-VP1-S	2426–2445	TGTAGTCCCAGCAAGCACAC	Forward	This study
EV-C99-VP1-A	3562–3583	CATGTACACCTGGCGATTTG	Reverse	This study
